# Supplementary material for: Physiological and Molecular Modulations to Drought Stress in the Brassica Species
Source: Int J Mol Sci. 2024 Mar 14;25(6):3306. doi: 10.3390/ijms25063306 (PMC10969959; doi:10.3390/ijms25063306)
Supplement: Supplementary file 1 [file ijms-25-03306-s001.zip › ijms-2894549-supplementary.pdf]

Supplementary Information

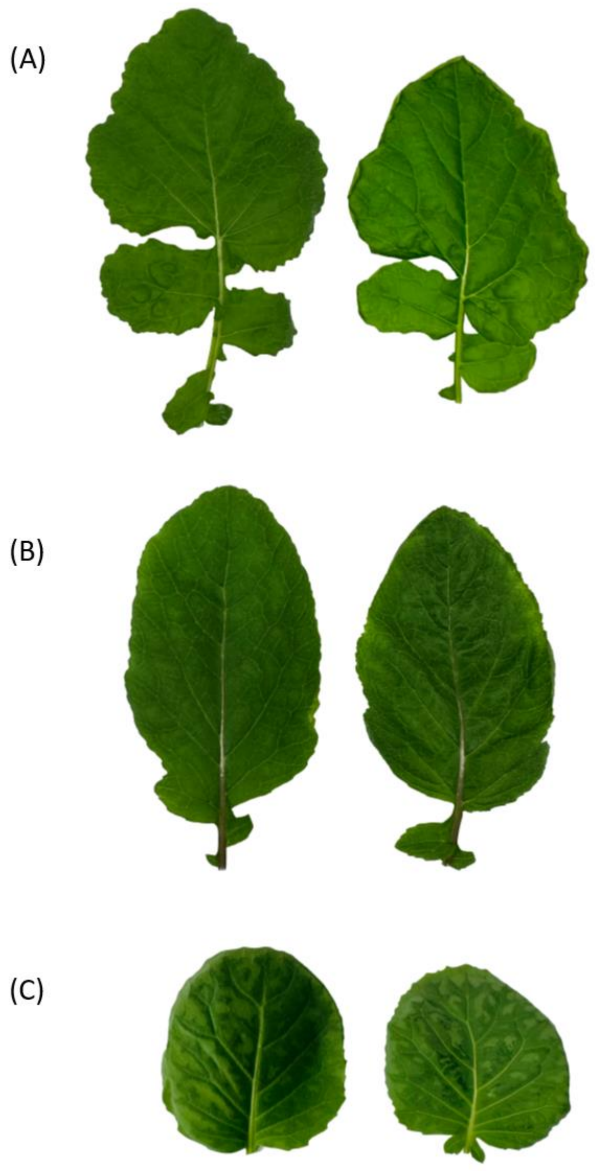

Figure S1. Comparison of leaves collected from control (left) and drought (right) conditions. (**A**) *B. rapa*, (**B**) *B. nigra*, (**C**) *B. oleracea.* Leaves from drought condition have white spots and rolled margins.

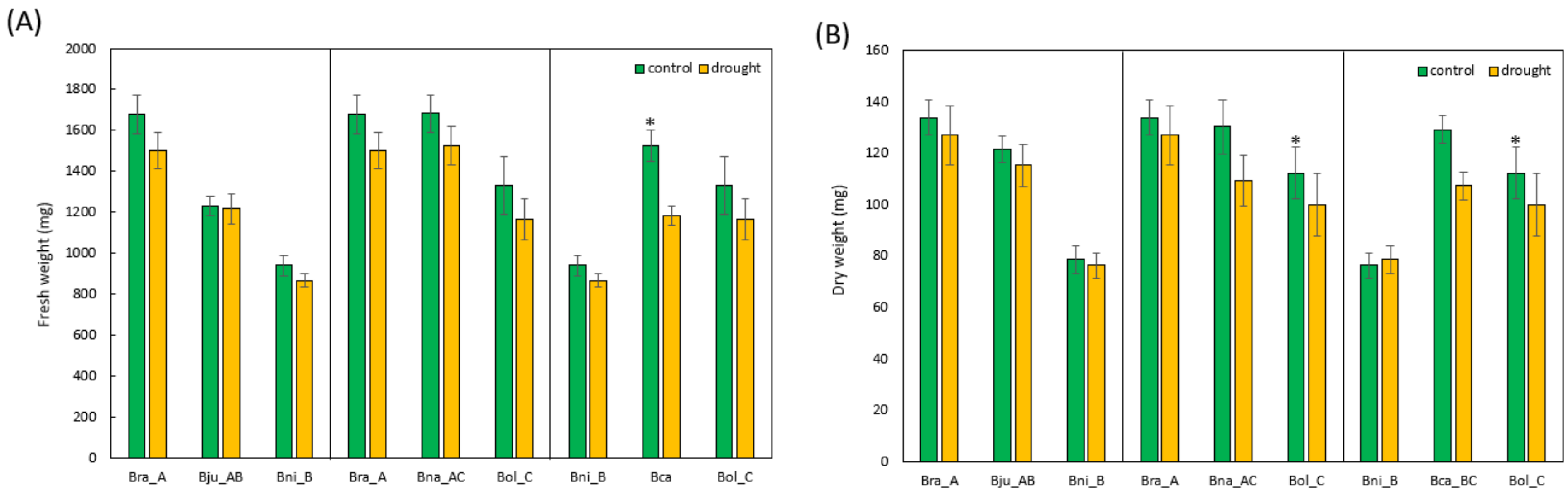

Figure S2. Comparison of fresh weight (**A**) and dry weight (**B**) between control and drought-stressed plants of *Brassica* species. Data were presented as mean ± standard error. Bra_A = *B. rapa* (*A*), Bju_AB = *B. juncea* (*AB*), Bni_B = *B. nigra* (*B*), Bna_AC = *B. napus* (*AC*), Bol_C = *B. oleracea* (*C*), Bca_BC = *B. carinata* (*BC*). Diploid species data were presented twice to compare the values of polyploids relative to those of their diploid parents. * = p-value < 0.05.

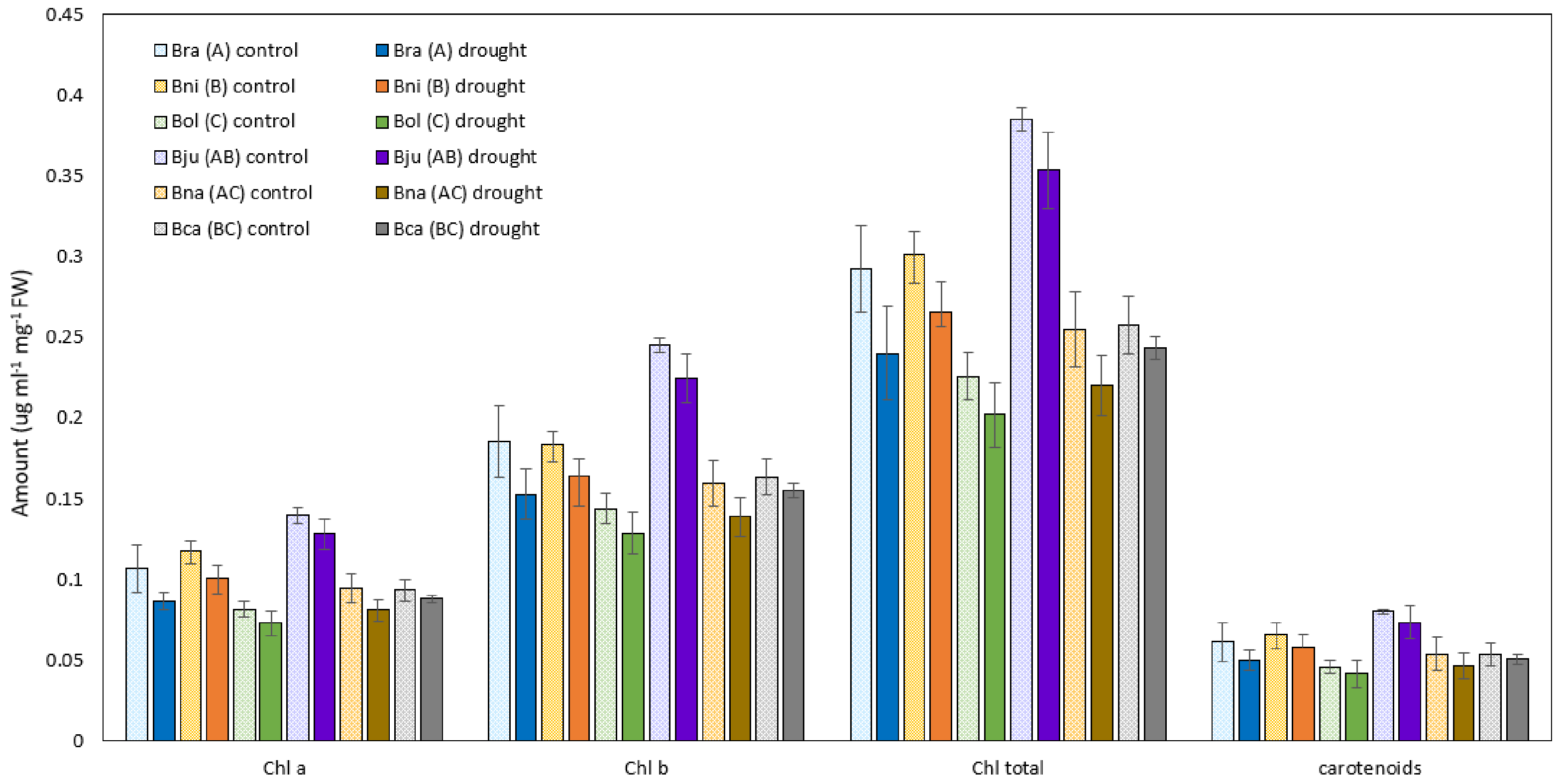

Figure S3. Comparison of pigment amounts between control and drought-stressed plants of *Brassica* species. Data were presented as mean ± standard error. Chl a = chlorophyll *a*, Chl b = chlorophyll b, Chl total = total chlorophyll. Bra (A) = *B. rapa* (*A*), Bju (AB) = *B. juncea* (*AB*), Bni (B) = *B. nigra* (*B*), Bna (AC) = *B. napus* (*AC*), Bol (C) = *B. oleracea* (*C*), Bca (BC) = *B. carinata* (*BC*).

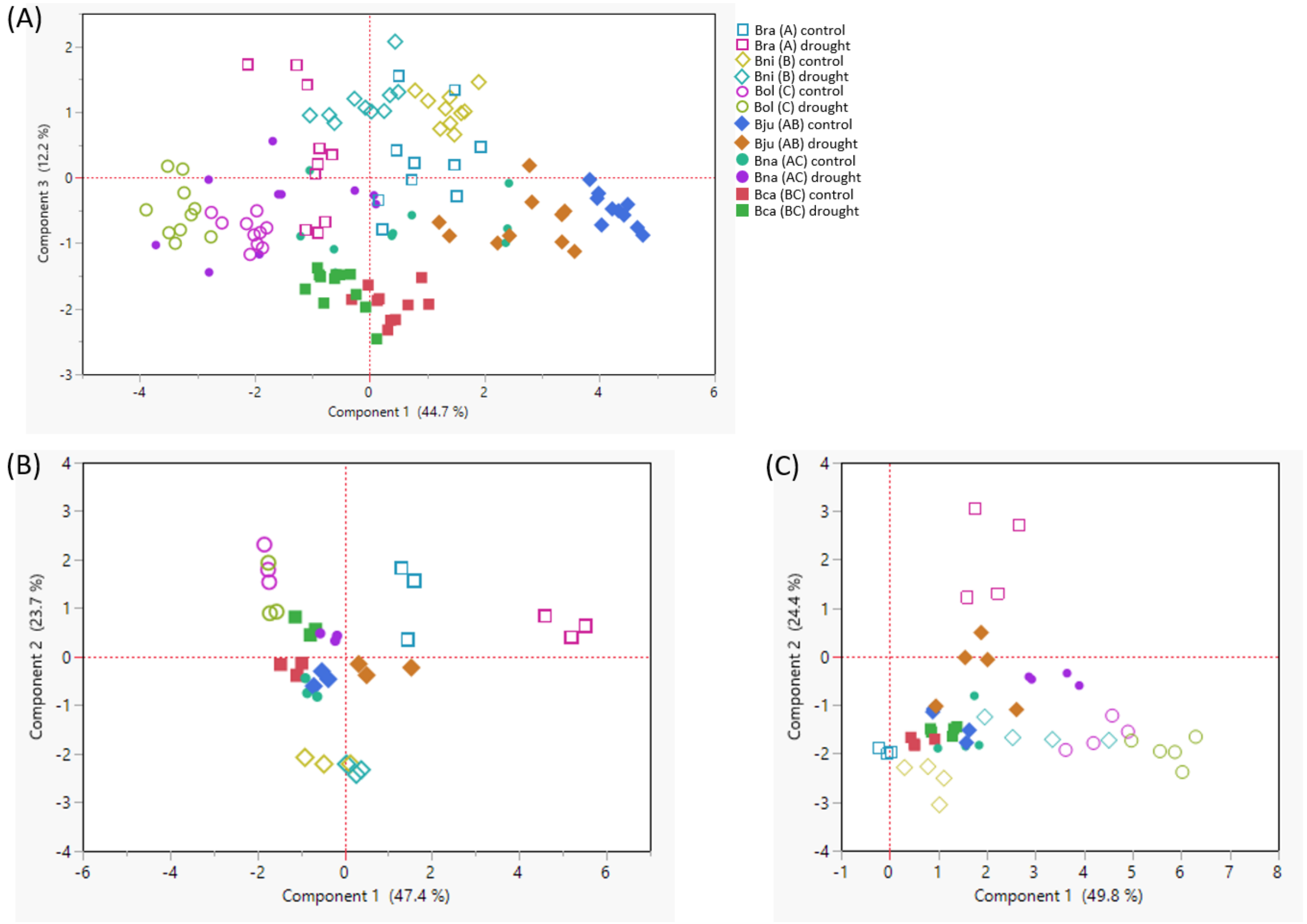

Figure S4. The PCA results of (**A**) morpho-physiological traits, (**B**) the expressions of seven genes, and (**C**) metabolite abundance. (**A**) Component 1 includes mostly pigment data (chlorophyll *a* and *b*, total chlorophyll, and carotenoids), while component 3 contains MDA and superoxide data. (**B**) Component 1 includes the expressions of *FSD6*, *CAT3*, *ERD1*, and *LEA4*, while component 2 contains the expressions of *MSD2*, *CSD1*, *ERD14*, and *ERD15*. (**C**) Component 1 includes three amino acids and GABA, while Component 2 contains PFOS and ABA.

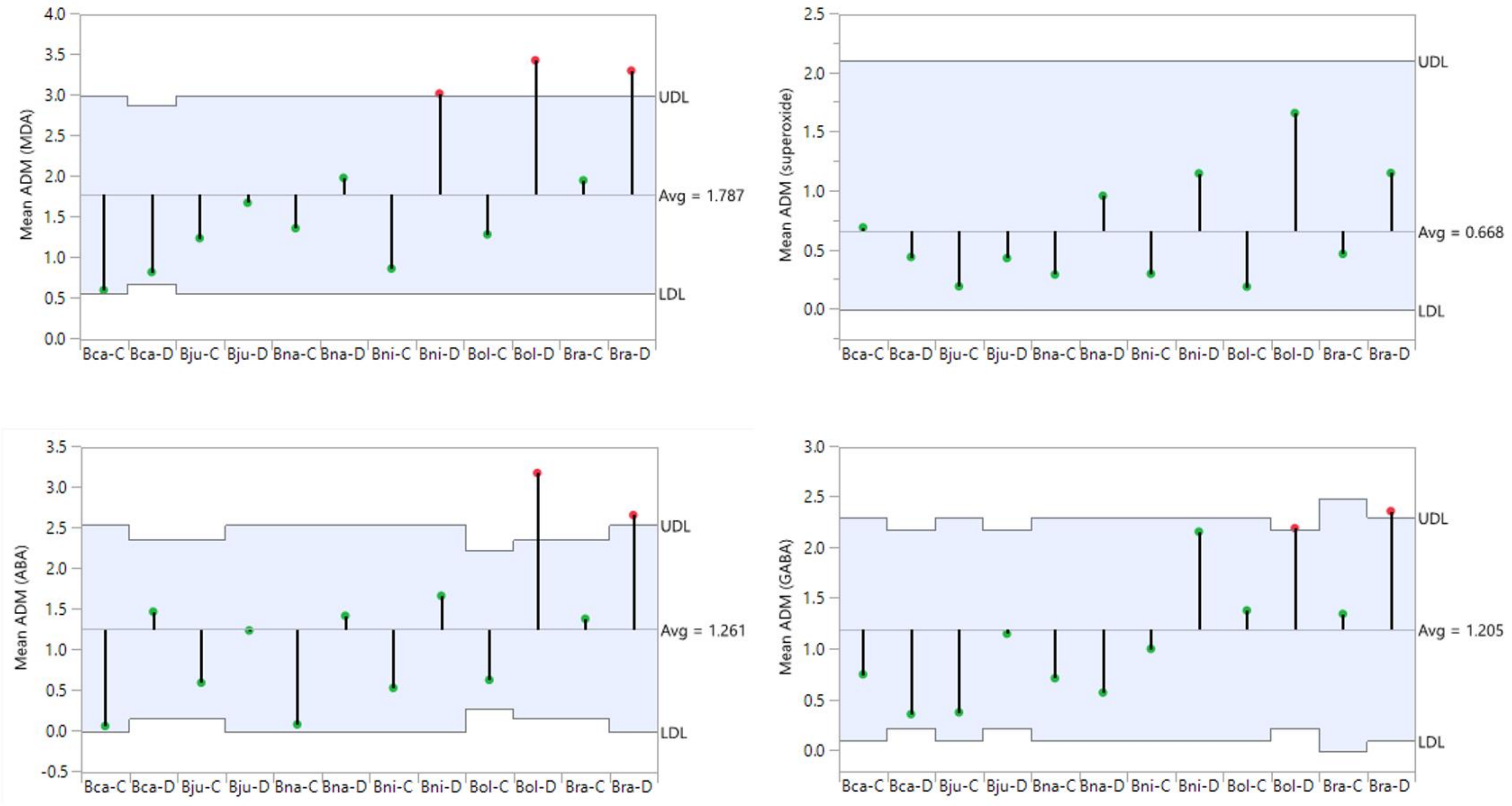

Figure S5. The results of Analysis of Means for Variances-Levene (ADM) of MDA, superoxide, ABA, and GABA data in six *Brassica* species. Red dots indicate significantly higher variance compared to other species ($\alpha = 0.05$). UDL = upper decision line, LDL = lower decision line, Avg = average of sample variance.

Table S1. The result of two-way ANOVA.

		Species	Treatment	Species*Treatment
Morpho-physiological traits	FW	<0.0001	0.1482	0.2759
	DW	<0.0001	0.9185	0.5036
	RWC	<0.0001	<0.0001	0.0500
	MDA	<0.0001	<0.0001	0.0040
	H_2O_2	<0.0001	<0.0001	<0.0001
	Superoxide ($O_2{\cdot}^-$)	<0.0001	<0.0001	<0.0001
	Chlorophyll *a*	<0.0001	<0.0001	0.0045
	Chlorophyll *b*	<0.0001	<0.0001	0.0317
	Total chlorophyll	<0.0001	<0.0001	0.0576
	Carotenoid	<0.0001	<0.0001	0.0382
Gene transcripts	*FSD6*	<0.0001	<0.0001	<0.0001
	MSD2	<0.0001	<0.0001	<0.0001
	CAT3	<0.0001	0.0001	0.0001
	CSD1	<0.0001	0.0002	0.0009
	ERD1	<0.0001	0.0001	0.1406
	ERD14	<0.0001	<0.0001	<0.0001
	ERD15	<0.0001	<0.0001	<0.0001
	LEA4	<0.0001	<0.0001	<0.0001
Metabolites	PFOS	n/a	n/a	0.0249
	proline	<0.0001	<0.0001	0.0243
	GABA	0.0001	0.0043	0.0122
	ABA	<0.0001	<0.0001	<0.0001
	phenylalanine	<0.0001	<0.0001	<0.0001
	tryptophan	<0.0001	<0.0001	0.0726
	leucine	<0.0001	0.1820	0.0385

Table S2. The information of *Brassica* species used in this study.

Species	Ploidy	Genome	Accession No.	Plant Name	Geographic origin
B. rapa	2x = 20	*AA*	PI 649186	Opava	Czech Republic
B. nigra	2x = 16	*BB*	PI 633151	G073	Ionian Islands, Greece
B. oleracea	2x =18	*CC*	PI 662840	Penca da Chaves	Portugal
B. juncea	4x = 36	*AABB*	PI 649124	Ames 26160	Iowa, USA
B. napus	4x = 38	*AACC*	PI 531277	Darmar	France
B. carinata	4x = 34	*BBCC*	Ames 2779	S.23	Ethiopia

Table S3. The primer information used for qRT-PCR.

Gene	Description	Primer sequences	Amplicon size (bp)
ACT1	actin 1, Internal control	F: TACATCACTGGGTACCAGGC<br>R: GAGGGCATCGATGTGGTTGT	117
CAT3	catalase 3	F: GGATCCTTACAAGTATCGGC<br>R: GGATAGGACCTCTGGGTC	118
CSD1	copper/zinc superoxide dismutase 1	F: CGTATCACCGGTCTCACAC<br>R: CGCTCCGTGTGTCATGTTGT	120
ERD1	early responsive to dehydration 1	F: ACAAGACTTGAAGTCGAGGC<br>R: GTTCTACGTAGTGGTCGTG	123
ERD14	early responsive to dehydration 14	F: GAGAAGCTTCCAGGACAC<br>R: GCTTCTCCTTAATCTTCTCC	136
ERD15	early responsive to dehydration 15	F: TGGCGATGGTTTCAGGAAG<br>R: GGTCGTCACCAACTGCCAC	116
LEA4	late embryogenesis abundant 4	F: ATGGACAAGGCCAAAGATG<br>R: AGGTGTTCTTGTTCATGCCG	130
FSD6	iron superoxide dismutase 6, chloroplastic	F: CCATCAGGAGAGCTTCTTGC<br>R: GCGTAAGCAAGCCAGGCC	122
MSD2	manganese superoxide dismutase 2, mitochondrial	F: CTAATTACAACAATGCCCTCG<br>R: TGGTTCACATGACCTCCGC	115
